# Dopamine Boosts Motivation for Prosocial Effort in Parkinson's Disease

**DOI:** 10.1523/JNEUROSCI.1593-24.2024

**Published:** 2025-01-02

**Authors:** Jamie Talbot, Jo Cutler, Marin Tamm, Simon J. Little, Catherine J. Harmer, Masud Husain, Patricia L. Lockwood, Matthew A. J. Apps

**Affiliations:** ^1^Centre for Human Brain Health, School of Psychology University of Birmingham, Birmingham B15 2TT, United Kingdom; ^2^Institute for Mental Health, School of Psychology, University of Birmingham, Birmingham B15 2TT, United Kingdom; ^3^Department of Experimental Psychology, University of Oxford, Oxford OX2 6GG, United Kingdom; ^4^Department of Neurology, University of California, San Francisco, California 94143; ^5^Department of Psychiatry, University of Oxford, Oxford OX3 7JX, United Kingdom; ^6^Wellcome Centre for Integrative Neuroimaging, University of Oxford, Oxford OX3 9DU, United Kingdom; ^7^Nuffield Department of Clinical Neurosciences, University of Oxford, Oxford OX3 9DU, United Kingdom

**Keywords:** dopamine, effort-based decision-making, motivation, Parkinson's disease, prosocial motivation

## Abstract

Being willing to exert effort to obtain rewards is a key component of motivation. Previous research has shown that boosting dopamine can increase the willingness to choose to exert effort to obtain rewards for ourselves. Yet often we must choose whether to exert effort, not for our own immediate benefit, but to be prosocial and obtain a benefit for someone else. Pharmacologically increasing dopamine availability has been shown to change social behaviors in experimental tasks, and dopamine degeneration in Parkinson's disease (PD) impacts a range of sociocognitive processes. However, the neuromodulators involved in deciding whether to exert effort to benefit others are unknown. Does dopamine modulate the willingness to exert prosocial effort? Here, male and female PD patients (*n* = 37) ON or OFF their dopaminergic medication completed a task where they chose whether to put in effort for larger reward, or rest and receive a smaller reward, on separate trials either to benefit themselves (“self”) or an anonymous other person (“other”). PD patients were more willing to exert effort to benefit themselves than another person, a pattern also observed in an age- and gender-matched control group (*n* = 42). However, crucially PD patients had increased willingness to exert effort for other relative to self, ON compared with OFF medication. These results suggest that dopamine augmentation in PD can increase levels of prosocial motivation, highlighting a key role for dopamine in motivation beyond obtaining rewards for ourselves.

## Significance Statement

Prosocial behaviors—acts that benefit other people—are fundamental for societal cohesion. Often prosocial acts, such as helping a friend move home, are effortful. However, the neurochemicals involved in choosing to put effort into prosocial acts are unknown. Dopamine is involved in motivating people to exert effort to obtain themselves rewards, but does it also make us choose to put more effort into prosocial behaviors? We find that dopamine-depleted Parkinson's disease patients are more willing to choose to put effort into prosocial acts ON dopamine-boosting medication compared with OFF. These results provide the first insight into the neurochemicals underlying prosocial effort and highlight dopamine as key to working hard to help others.

## Introduction

Being willing to exert effort for reward is key component of motivation linked to health and well-being (Le Heron et al., 2017; Husain and Roiser, 2018). Research in animal models, healthy humans, and Parkinson’s disease (PD)—a disorder characterized by dopaminergic degeneration—has shown that boosting or depleting dopamine can lead to increases or decreases in the willingness to exert effort to obtain ourselves rewards (Salamone et al., 2007; Chong et al., 2015; Le Heron et al., 2017; Pessiglione et al., 2018; McGuigan et al., 2019; Westbrook et al., 2020). However, often we have to make decisions about whether we are motivated and willing to exert effort to be prosocial and obtain rewards for others without an immediate benefit for ourselves (Lockwood et al., 2017, 2022). However, little is known about the neuromodulators that underlie this “prosocial motivation.”

Parkinson's disease (PD) is typically characterized as a movement disorder (Forno, 1996) attributed to dopaminergic degeneration. However, patients also exhibit a range of nonmotor symptoms (Schapira et al., 2017) that include changes in social cognition and behavior. Indeed, experimental tasks have revealed a reduced ability to perceive and understand social stimuli (Kawamura and Koyama, 2007; Narme et al., 2013; Pell et al., 2014). Research has begun to examine whether dopamine depletion in PD may alter prosocial tendencies with economic games that measure sensitivity to one's own and other people's financial rewards. However, the results are mixed. PD patients have been shown to be more impulsively generous (Amstutz et al., 2021), while others have suggested only limited differences in how willing patients are to be prosocial compared with controls (Sparrow et al., 2021). Research in healthy participants is similarly inconclusive, with higher dopamine levels associated with a prosocial desire for equality (Sáez et al., 2015), but also reductions in generosity (Artigas et al., 2019) and an increased willingness to earn financial rewards at the expense of physical pain to others (Crockett et al., 2015).

While economic games have been fruitful for understanding prosocial tendencies, the limitations of existing approaches have not allowed us to learn what role, if any, dopamine has in prosocial behavior. Specifically, as the tasks often focus solely on trading off financial rewards for self versus other, they ignore the fact that prosocial behaviors often require effort (Contreras-Huerta et al., 2020). In addition, existing paradigms can confound sensitivity to one's own and others’ rewards (Lockwood et al., 2020). In contrast, researchers have developed paradigms that can measure prosocial motivation—the willingness to exert effort to obtain rewards for others (Lockwood et al., 2017; Contreras-Huerta et al., 2022; Forbes et al., 2023). These studies have shown that while people will exert effort for other's benefit, they are less willing to do so compared with when they will obtain the reward themselves. Moreover, decisions for self and other are associated with at least some neuroanatomical differences in the systems that guide them, with evidence of specialization for prosocial effort processing in the anterior cingulate gyrus and ventromedial prefrontal cortex (Lockwood et al., 2022, 2024).

**Figure 1.**
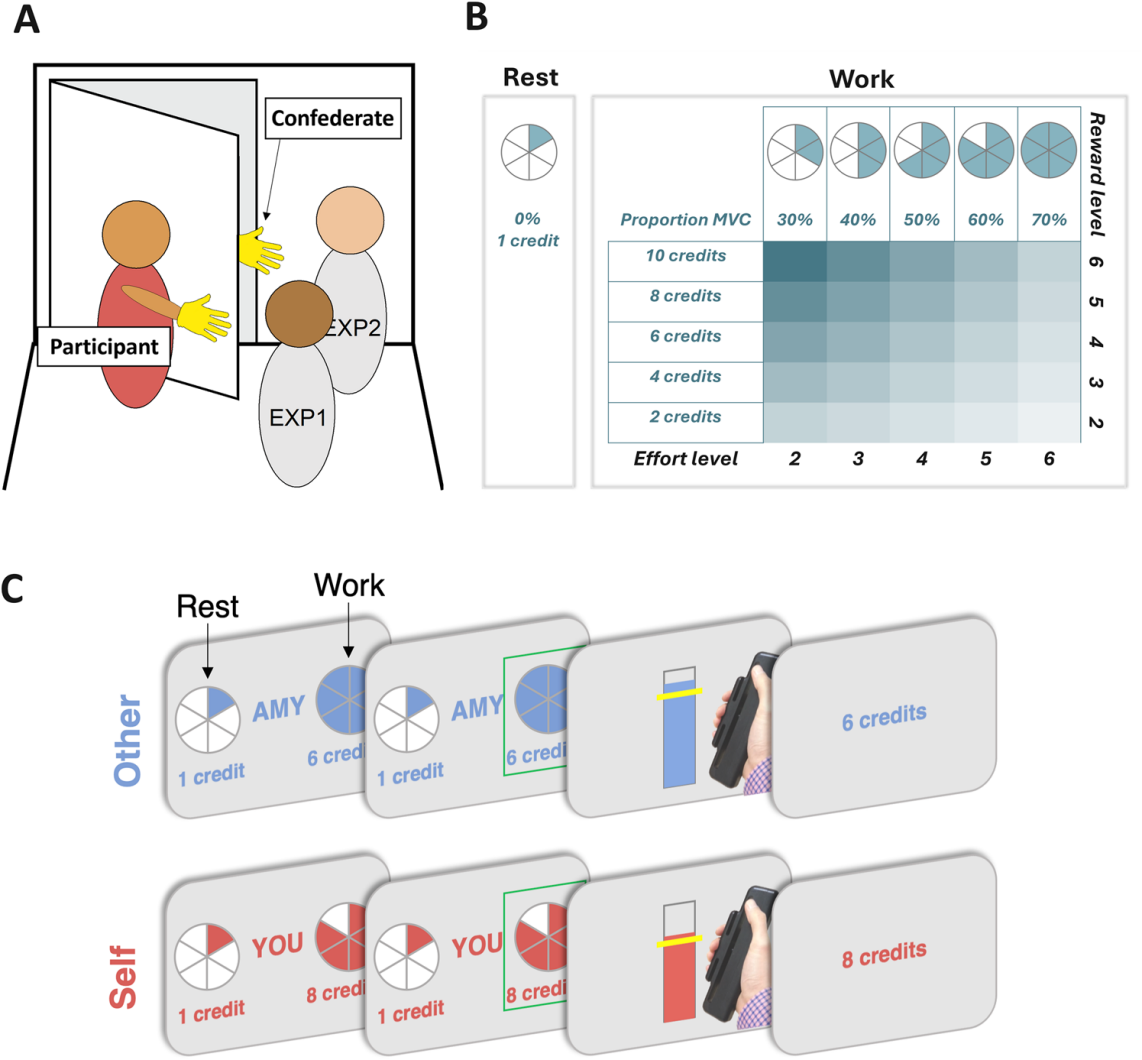
***A***, To ensure participants believed they were making decisions that benefitted a real other person, they first completed a role assignment procedure. Participants were designated as “Player 1” (self) and told that they would be making decisions that impacted another player “Player 2” (other) who they met at the beginning of the testing session with their identity obscured. The procedure involved four people, two experimenters, EXP1 and EXP2, and two participants, self and other. ***B***, Participants were presented on each trial with a choice between a rest option which required no effort (0% maximum voluntary contraction (MVC), corresponding to one segment of the pie chart) for a low reward of 1 credit, and a work option, which required more effort (30–70% MVC, corresponding to 2–6 segments in the pie chart) yet also generated more reward (2–10 credits). These effort and reward magnitudes are presented as levels between 2 and 6, with higher numbers representing greater effort/reward. The offered reward and effort levels were orthogonal in the design. ***C***, The experimental session began with participants being instructed to squeeze as hard as they could to measure their MVC on a handheld dynamometer to threshold each effort level to their own strength. After thresholding and practice, participants were asked to choose between resting for 1 credit, or exerting effort for greater reward, with effort and reward manipulated as described above. The recipient of the reward (“you”/self, or a common, gender-matched name assigned to Player 2/other) was displayed at each offer screen. After making their choice, participants then had to exert the required force to receive the reward. Visual feedback of the amount of force exerted was displayed on the screen. Participants were informed that they would have to reach the required force level (marked by the yellow line) for at least 1 s of a 3 s window. Participants then saw the outcome that corresponded to the offer they had chosen, unless they were unsuccessful, in which case “0 credits” was displayed. Crucially, on self-trials, participants made the choice, exerted the effort, and received the reward themselves, whereas on other trials (“AMY” in this example), participants made the choice and exerted the effort, but the other participant received the reward.

Here, we examined the role of dopamine in prosocial motivation by testing PD patients ON and OFF their prescribed dopaminergic medication in a counterbalanced order—as well as a group of age-matched controls—on a paradigm that measures the willingness to exert effort for self or other (Lockwood et al., 2017). Participants made choices between a no-effort, low-reward option and a high-effort, high-reward option, which varied independently on each trial in terms of effort and reward on offer (Fig. 1). On half the trials, participants made the choice, exerted the chosen effort, and received rewards which increased their own bonus payment. On the other half, they made the choice and exerted the effort, but the reward boosted the bonus payment of an anonymous other individual. Using this design, we could adjudicate between alternative hypotheses about whether dopamine might impact the motivation to exert effort to benefit self, other, or both.

## Materials and Methods

### Participants

Forty participants with Parkinson's disease were recruited via the University of Oxford Cognitive Neurology participant database. Inclusion criteria included a positive diagnosis of PD as per UK Brain Bank criteria and current treatment with levodopa. Exclusion criteria included significant coexistent neurological or psychiatric disease, significant cognitive impairment, or physical impairment which would otherwise limit performance on behavioral task. Two participants withdrew from the study after the first session, and one participant missed over 50% of trials and was excluded, leaving 37 patients (29 male) in the final analysis. Forty-two healthy, age-matched controls (29 male) were recruited who had no underlying neurological condition. All participants provided written informed consent. The study was approved by the Medical Sciences Division Research Ethics Committee of the University of Oxford. Participants were renumerated at a basic rate of £10 per hour, plus an additional performance-based payment of up to £5 based on credits won during the task. They were also told the number of credits earned in the prosocial condition would translate into an additional payment of up to £5 for the other participant.

### Questionnaire measures

To ensure participants included in this study were not cognitively impaired to a degree that might impact their understanding of the task instructions, participants undertook Addenbrooke's Cognitive Examination III (ACE-III; Table 1). Participants also completed validated screening questionnaires the Geriatric Depression Scale-15 (GDS-15), a rating scale for depression (Yesavage and Sheikh, 1986), and the Apathy Motivation Index (AMI; Ang et al., 2017) and Lille Apathy Rating Scale (LARS; Sockeel et al., 2006) which quantify symptoms of apathy and the Questionnaire of Cognitive and Affective Empathy (QCAE; Reniers et al., 2011), a score measuring cognitive and affective dimensions of empathy. We note that no clear results were found relating these measures to task behavior in patients ON or OFF medication, and so none are reported in detail in the results. To examine PD symptom severity, patients also completed the Unified Parkinson's Disease Rating Scale (UPDRS).

**Table 1.** Demographic information and mean/standard deviations for questionnaire scores in each group

	Healthy controls	Parkinson's disease	Contrast
Age	69.7 ± 5.7	67.9 ± 6.7	*t* = 1.27, *p *= 0.21
Male sex	29 (69.0%)	29 (78.4%)	*χ*^2^ = 12.18, *p *= 1
Right-handed	35 (83.3%)	33 (89.2%)	*χ*^2^ = 20.85, *p *= 1
ACE total	96.5 ± 2.6	95.2 ± 4.4	*W* = 883, *p *= 0.30
AMI total	1.1 ± 0.4	1.2 ± 0.4	*t* = −0.83, *p *= 0.41
Behavioral	1.0 ± 0.6	1.3 ± 0.6	
Emotional	1.1 ± 0.5	0.9 ± 0.5	
Social	1.3 ± 0.8	1.4 ± 0.7	
GDS total	1.1 ± 1.4	2.7 ± 3.0	*W* = 534, *p *= 0.013
Depression	0.6 ± 1.0	1.5 ± 2.0	*W* = 542, *p *= 0.012
Apathy	0.5 ± 0.8	1.1 ± 1.1	*W* = 544, *p *= 0.012
LARS total	−28.7 ± 3.0	−27.7 ± 4.6	*W* = 690, *p *= 0.50
Action initiation	−3.7 ± 0.5	−3.3 ± 0.8	
Intellectual curiosity	−2.5 ± 0.9	−3.1 ± 0.8	
Emotional	−3.4 ± 0.4	−2.9 ± 0.84	
Self-awareness	−2.8 ± 1.6	−3.0 ± 1.3	
QCAE total	87.8 ± 10.0	92.3 ± 11.1	*t* = −1.86, *p *= 0.067
Cognitive empathy	57.3 ± 7.6	58.4 ± 7.0	*t* = −0.66, *p *= 0.51
Affective empathy	30.5 ± 4.8	33.8 ± 6.0	*t* = −2.74, *p *= 0.0079
SRP total	41.5 ± 8.6	40.7 ± 11.3	*W* = 845, *p *= 0.39
UPDS III (off)	N/A	33.0 ± 12.9	ON versus OFF;
UPDRS III (on)	N/A	27.9 ± 11.5	*W* = 623, *p* < 0.001
Levodopa equivalent dose (mg/day)	N/A	454 ± 224	N/A

Contrasts between groups use the appropriate between-subjects test, either *t*, *t* test for data meeting normality assumptions; *W*, Wilcoxon rank sum test for continuous data violating assumptions; *χ*^2^, chi-square test for binary data. AMI, Apathy Motivation Index; ACE-III, Addenbrooke’s Cognitive Examination III; GDS, Geriatric Depression Scale; LARS, Lille Apathy Rating Scale; QCAE, Questionnaire of Cognitive and Affective Empathy; SRP, Self-Report Psychopathy Scale; UPDRS, Unified Parkinson's Disease Rating Scale.

### Comparison between groups

PD and healthy control groups were not significantly different in terms of sex, age, cognitive ability, or years of education (Table 1). The Parkinson's group ranked significantly higher on questionnaire scores of depression, as is common in PD (Remy et al., 2005; Aarsland et al., 2011), but were not significantly more apathetic. Patients scored significantly higher in affective empathy than healthy controls (Table 1).

### Procedure

We examined whether dopamine deficiency in Parkinson's disease or enhancement of dopamine signaling via dopaminergic medication leads to differences in the willingness to exert effort to obtain rewards for self or an anonymous other person. To achieve this, we used an established paradigm (Lockwood et al., 2017, 2021, 2022) that measures how willing people are to exert physical effort (grip force) to reward self or other and tested a group of Parkinson's disease patients who were taking prescribed dopaminergic medication. PD patients were tested on both ON and OFF medication in a counterbalanced within-subject design and compared with an age-matched cohort of healthy controls.

We followed a protocol that has previously proved successful in examining the effects of dopamine medication on motivation in PD (Chong et al., 2015; Le Heron et al., 2018). For the “ON” session, patients were instructed to take their usual medication on the morning of testing, while for the “OFF” session, the same patients were instructed to omit all medications with effects on dopamine transmission on the morning of testing. Prior to testing, patients had provided a list of their medications and were instructed which they needed to stop [these included the dopamine precursor levodopa in 37 (100%) patients, the dopamine agonists ropinirole and pramipexole in 12 (32%) patients, and the monoamine oxidase-B inhibitor rasagiline in 12 (32%) patients]. Participant testing sessions always began between 8:30 and 10:30 in the morning. Thus, patients would be between 8 and 14 h since their last dose in the off session but <4 h during the on session (and therefore dopamine would be close to peak plasma levels; Hauser, 2009). Adherence to the protocol was confirmed with patients on the day, with all reporting satisfactory adherence and UPDRS part III (motor exam) significantly improved in “ON” versus “OFF” conditions (Wilcoxson rank sum *p* < 0.001). Session order was randomized and counterbalanced in participants with PD. Healthy participants were only tested in one session as they were not taking dopaminergic medication. In all sessions, participants and patients completed the task, followed by additional tasks to be reported in separate publications.

### Prosocial effort task

Participants made a series of decisions between two options: a “work” offer that was higher in physical effort but obtained greater reward and a “rest” offer that was equal in duration but involved no physical effort and obtained a lower reward (Fig. 1*B*). On half of the trials, the participants made these decisions and exerted the effort that they chose, and the rewards obtained increased their own bonus payment proportionally. On the other half, the participants were presented with the same offers and exerted the same type of effort but were instructed that rewards earned would be delivered to an anonymous other participant. Thus using this design, we could measure how willing people were to work versus rest for self and other.

Physical effort was operationalized as the amount of force participants exerted on a handheld dynamometer. On arrival and after consent, participants completed a calibration phase to estimate their maximum voluntary contraction (MVC). They were instructed to squeeze the handheld dynamometer as hard as they could during a 5 s window. Participants were provided with visual feedback while doing so of a red fill dynamically moving proportionally to their grip force up and down a white bar. Following this, they performed two further trials and were instructed to squeeze hard enough to reach a line that was set as 110% of the previous maximum on the first and 105% on the second. The maximum reached during this whole procedure—their maximum voluntary contraction (MVC)—was used to calibrate grip force levels of the experiment. Effort levels in the main experiment were between 30 and 70% of this MVC, in 10% increments. To succeed at an effort level in the main experiment, participants had to match or exceed the required percentage for a total of 1 s out of a 3 s period. After this calibration procedure, and before any task instructions, participants were introduced to another participant anonymously (see below, Role assignment). Before the main task started, participants practiced each of those five effort levels three times in ascending sequence and were awarded 1 credit if successful or 0 credits if not, to become familiar with the effort required at each level of force.

Next, participants were required to choose between one of two offers on each trial. One option allowed participants to earn a low reward but required no effort (rest). The other presented a variable higher-reward, higher-effort offer (work) of the same duration. The low-reward, no-effort offer earned 1 credit and required no effort but offered 3 s of rest. The higher-reward, higher-effort offers varied from 2 to 10 credits (in 2-credit increments). Effort ranged from 30 to 70% (in 10% increments) of the participants’ MVC. Participants were instructed that they could win a bonus of up to £5 and that more credits earned corresponded to a greater bonus, but were not made aware of the exchange rate while completing the task to ensure that they did not try to compute a running total. Critically, each trial also varied in whether the outcome would be delivered to the participant themselves (“self”) or the receiver participant (“other,” prosocial). The level of effort required for each offer was represented using colored portions of a pie chart: blue for other and red for self (Fig. 1*C*). Rewards (credits) on offer for each option were written in color below. Participants were allotted 3.5 s to make a choice between the rest and work offers. If they failed to choose an option, they were awarded 0 credits after a full trial duration. After choosing, participants were shown a screen with a yellow horizontal bar on an empty vertical box. The horizontal bar represented the level of effort required; the box was filled according to the force participants exerted on the dynamometer, providing feedback in real time on the color of who would receive the rewards. For a trial to be considered successful, and rewards obtained, participants had to accumulate at least 1 s at or above the required force level across the 3 s force period.

There were 150 trials in total, 75 “self” and 75 “other” trials. Each of the 25 effort–reward combinations was presented three times for each agent. The task was separated into three blocks of 50 trials each with extended breaks offered in-between blocks at the participants’ discretion to minimize the effects of fatigue (Müller et al., 2021).

### Role assignment

To ensure participants believed their effort would influence the bonus payment of another person, but that this person would remain anonymous, participants completed a role assignment procedure (Lockwood et al., 2022). Participants were instructed not to speak and wore a glove to hide any physical characteristics and to ensure they were anonymous to one another (Fig. 1*A*). A second experimenter brought the confederate to the other side of a door who was also instructed not to speak and wore a glove. Participants only ever saw the gloved hand of the confederate, but they waved to each other to make it clear there was another person there. The experimenter tossed a coin to determine who picked a ball from the box first and then told the participants which roles they had been assigned to, based on the ball that they picked. Unbeknownst to participants, our procedure ensured that participants always ended up in the role of the person performing the effort task and they were led to believe the other participant would be performing separate tasks in another room. We emphasized that the other participant would only perform experimental tasks that would result in outcomes for themselves and would be unaware of the task performed by the other participant, so any reward given would be anonymous. They would also never be introduced or told who the other participant was to minimize any effects of reciprocity. In patients attending the second testing session, the role selection process was repeated with participants informed they would be playing in tandem with a new participant to minimize any inequity aversion arising from the first session. We revealed the first name of the other participant that was always a common name matched to the gender of the participant performing the experiment, to further emphasize the recipient of rewards on “other” trials while at the same time minimizing the potential for bias (e.g., based on gender or prior personal association).

After finishing the task, participants completed a short debriefing questionnaire where they were probed as to whether they believed they were earning rewards for another participant. No participant reported a disbelief in the deception.

### Statistical analysis

All analyses were carried out using R (The R Foundation, version 4.1). For each outcome (choices, force exerted), we ran (generalized) linear mixed-effects models (GLMM) starting with a hypothesis-driven maximal model (Barr et al., 2013; Matuschek et al., 2017). This was defined based on the main experimental hypotheses; that Parkinson's disease or levodopa therapy would influence prosocial motivation, potentially via enhancement of reward sensitivity or effort aversion. Separate models compared patients “ON” and “OFF” medication (within-subjects), unmedicated patients to healthy controls, and medicated patients to healthy controls. The maximal models therefore contained main effects and included all possible (up to three-way) interactions of reward:recipient:group and effort:recipient:group. Between-subjects models (“group” is HC vs PD) included random slopes of reward, recipient, reward:recipient, effort, and effort:recipient varying by subject and included a subject-level intercept. Within-subjects models (“group” becomes “drug”: PD patients “ON” vs “OFF”) also included three-way interactions of reward:recipient:drug and effort:recipient:drug, to account for subject-level variance caused by drug effects. Using the packages *lme4* and *buildmer*, a model for each outcome was selected by ensuring convergence and then removing effects by backward stepwise elimination based on change in log likelihood. Fixed effects were specified to remain constant in the model with only the random effects refined. Thus, in each case, a parsimonious model was selected that tested the main experimental hypotheses while allowing for adequate subject-dependent variance of the main experimental variables.

All numeric variables were scaled and mean-centered, and the “recipient” variable was factor-coded with sum-to-zero contrast. The “group” (or “drug”) variable was dummy-coded, with healthy controls and the unmedicated state reflecting the reference group. Statistical reporting of linear mixed models includes estimated odds ratios with *p*-values based on Wald *z* scores (*summary* function in R's lmerTest package). Post hoc pairwise comparisons (estimated marginal means/least squares) are calculated using R's *emmeans* package. Analysis of choices used the logit link function for binomial data and effort was quadratically transformed in line with previous studies (Lockwood et al., 2021).

While not a primary hypothesis, we noted during exploration of the data that participants’ behavior changed over trials, and thus inclusion of trial number in models (incorporating groupwise interactions between effort, reward, and recipient) resulted in significantly improved model fit (on basis of AIC and BIC criteria). We include these results as exploratory analyses to provide insights into dynamic choice behavior including potential effects of fatigue.

Below are the specifications of linear models selected by stepwise elimination

#### PD OFF versus PD ON

GLMM for choice:

Choice ∼ 1 + Drug + Effort + Reward + Recipient + Drug:Recipient + Drug:Reward + Recipient:Reward + Drug:Effort + Recipient:Effort + Drug:Recipient:Effort + Drug:Recipient:Reward + (1 + Reward + Effort + Recipient + Drug + Drug:Effort)

GLMM for choice (incorporating session):

Choice ∼ 1 + Drug + Effort + Reward + Recipient + Session + Drug:Recipient + Drug:Reward + Recipient:Reward + Drug:Effort + Recipient:Effort + Drug:Session + Recipient:Session + Reward:Session + Effort:Session + Drug:Recipient:Reward + Drug:Recipient:Session + Drug:Reward:Session + Recipient:Reward:Session + Drug:Recipient:Effort + Drug:Session:Effort + Recipient:Session:Effort + Drug:Recipient:Reward:Session + Drug:Recipient:Effort:Session + (1 + Recipient + Effort + Reward + Drug + Drug:Effort + Drug:Reward | ID)

GLMM for choice (incorporating trial number):

Choice ∼ 1 + Drug + Effort + Reward + Recipient + Trial number + Drug:Recipient + Drug:Reward + Recipient:Reward + Drug:Effort + Recipient:Effort + Drug:Trial number + Recipient:Trial number + Effort:Trial number + Drug:Effort:Trial number + Reward:Trial number + Drug:Recipient:Reward + Drug:Recipient:Effort + Drug:Recipient:Trial number + Drug:Reward:Trial number + (1 + Reward + Effort + Recipient + Drug + Trial number + Drug:Trial number + Drug:Reward + Drug:Recipient | ID)

LMM for force:

Force ∼ 1 + Drug + Effort + Reward + Recipient + Drug:Recipient + Drug:Reward + Recipient:Reward + Drug:Effort + Recipient:Effort + Drug:Recipient:Effort + Drug:Recipient:Reward + (1 + Effort + Reward + Recipient + Drug + Drug:Effort + Drug:Reward | ID)

#### HC versus PD OFF

GLMM for choice:

Choice ∼ 1 + Group + Effort + Reward + Recipient + Group:Recipient + Group:Reward + Recipient:Reward + Group:Effort + Recipient:Effort + Group:Recipient:Effort + Group:Recipient:Reward + (1 + Reward + Effort + Recipient + Recipient:Reward | ID)

GLMM for choice (incorporating trial number):

Choice ∼ 1 + Group + Effort + Reward + Recipient + Trial number + Group:Recipient + Group:Reward + Recipient:Reward + Group:Effort + Recipient:Effort + Group:Trial number + Recipient:Trial number + Effort:Trial number + Group:Effort:Trial number + Reward:Trial number + Group:Recipient:Reward + Group:Recipient:Effort + Group:Recipient:Trial number + Group:Reward:Trial number + (1 + Reward + Effort + Recipient + Trial number +Reward:Trial number + Effort:Trial number | ID)

LMM for force:

Force ∼ 1 + Group + Effort + Reward + Recipient + Group:Recipient + Group:Reward + Recipient:Reward + Group:Effort + Recipient:Effort + Group:Recipient:Effort + Group:Recipient:Reward + (1 + Effort + Reward | ID)

#### HC versus PD ON

GLMM for choice:

Choice ∼ 1 + Group + Effort + Reward + Recipient + Group:Recipient + Group:Reward + Recipient:Reward + Group:Effort + Recipient:Effort + Group:Recipient:Effort + Group:Recipient:Reward + (1 + Reward + Effort + Recipient + Recipient:Reward | ID)

GLMM for choice (incorporating trial number):

Choice ∼ 1 + Group + Effort + Reward + Recipient + Trial number + Group:Recipient + Group:Reward + Recipient:Reward + Group:Effort + Recipient:Effort + Group:Trial number + Recipient:Trial number + Effort:Trial number + Group:Effort:Trial number + Reward:Trial number + Group:Recipient:Reward + Group:Recipient:Effort + Group:Recipient:Trial number + Group:Reward:Trial number + (1 + Reward + Effort + Recipient + Trial number + Reward: Trial number | ID)

LMM for force:

Force ∼ 1 + Group + Effort + Reward + Recipient + Group:Recipient + Group:Reward + Recipient:Reward + Group:Effort + Recipient:Effort + Group:Recipient:Effort + Group:Recipient:Reward + (1 + Effort + Reward + Recipient | ID)

## Results

Previous research suggests multiple alternative possible hypotheses for the role of dopamine in prosocial behavior. Boosting dopamine could (1) increase the willingness to exert effort for self (Chong et al., 2015), (2) increase the willingness to be prosocial (Sáez et al., 2015), (3) reduce the willingness to be prosocial (Crockett et al., 2015), or (4) be any combination of the previous three hypotheses. Moreover, this effect could impact either choices to exert effort or the actual energization of the effortful acts. We used a task that could allow us to adjudicate between these and examine how willing people were to exert effort for self and other (Lockwood et al., 2017, 2021, 2022). We tested 37 PD patients in a within-subject design ON and OFF their typical dopaminergic medication as well as an age-matched control group (*n* = 42).

### Levodopa leads to greater willingness to benefit others

**Figure 2.**
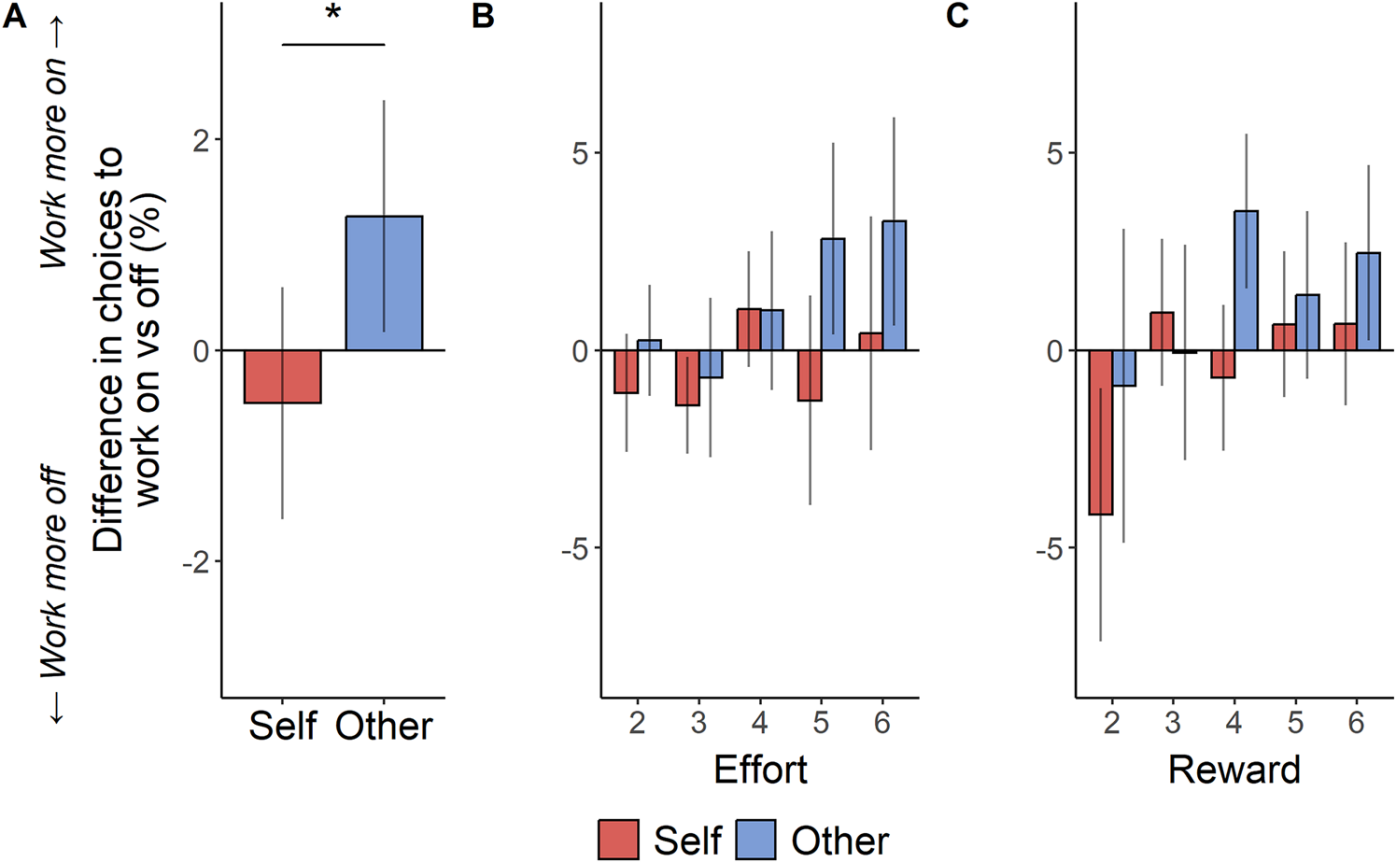
Dopamine medication increases motivation to exert effort to help another person relative to helping oneself in PD. ***A***, Mean difference between “ON” versus “OFF” in average acceptance of high-effort high-reward “work” offers split between self and other trials in PD patients. Positive bars represent greater acceptance ON versus OFF. The same difference is broken down into the different levels of (***B***) effort required and (***C***) reward available. Error bars represent SEM.

**Figure 3.**
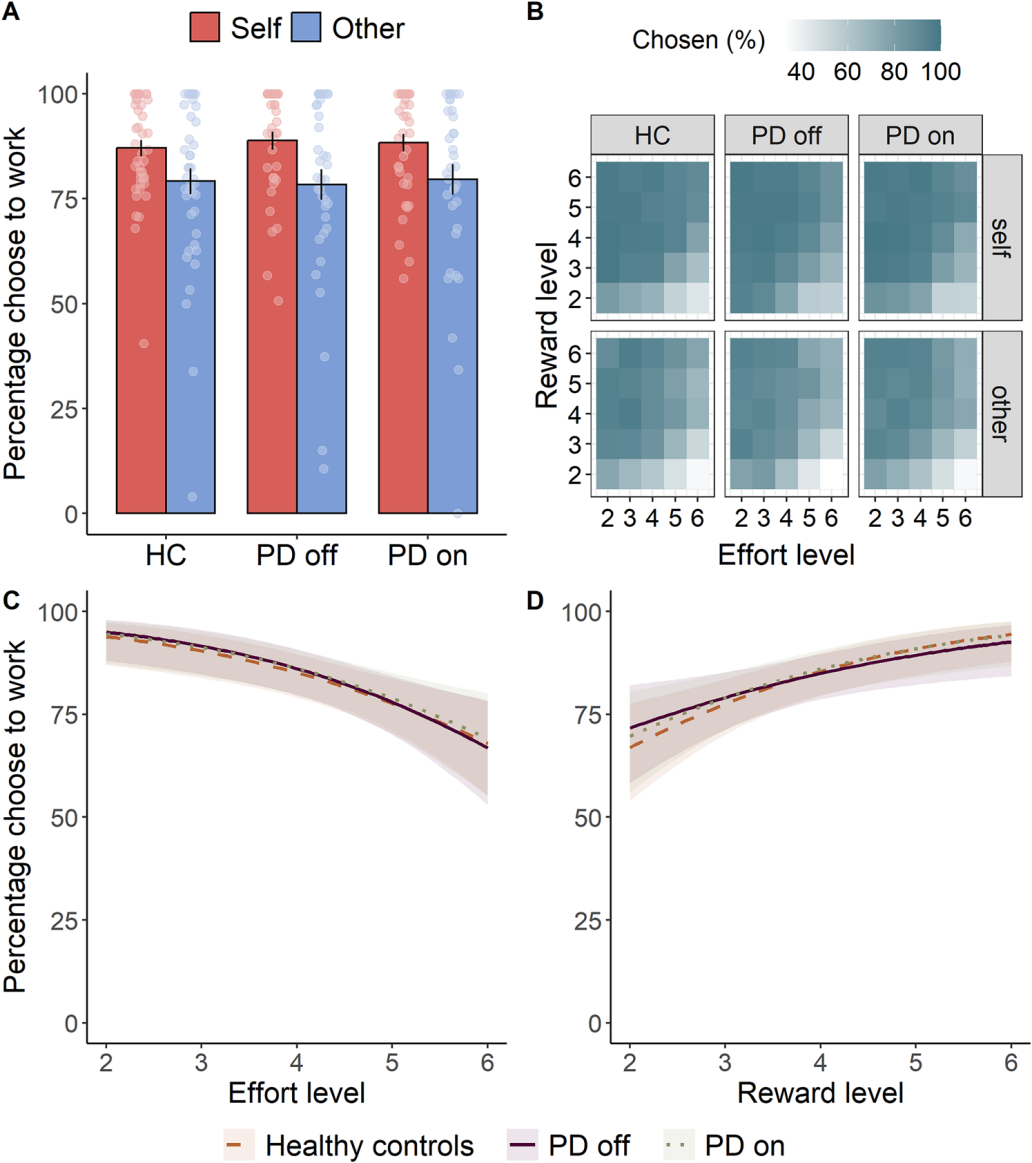
***A***, Mean proportion of accepted work offers in controls and patients. Healthy controls and patients both ON and OFF dopamine show greater willingness to exert effort when rewards are for themselves compared with another person. Errors bars represent SEM. ***B***, Heat map showing ordinary marginal means reflecting the percentage of work options chosen, collapsed across levels of reward (levels 2–6, reflecting 2–10 credits), effort (levels 2–6, reflecting 30–70% maximum voluntary contraction) and recipient (self and other) for the three groups (healthy controls, PD OFF, PD ON). All three groups display aversion to effort (***C***) and sensitivity to reward (***D***; displayed plots show GLM-estimated conditional means ± 95% confidence intervals collapsed over self and other).

First, we examined choice behavior within PD patients to assess the impact of dopaminergic medication. We ran a generalized linear mixed model (GLMM; see Materials and Methods) to test the effect of drug (ON/OFF dopamine medication) and how this interacted with the effort required (30–70% MVC), reward available (2–10 credits), and the recipient (self/other). We found that patients exerted less effort for other compared with self both ON and OFF medication [OR = 2.45, SE = 0.59, CI: (1.53, 3.91), *z* = 3.75, *p *< 0.001; Fig. 3*A*]. However interestingly, this difference between self and other was reduced ON medication compared with OFF [drug:recipient interaction OR = 0.74, SE = 0.09, CI: (0.59, 0.93), *z* = −2.59, *p *= 0.010; Fig. 2*A*]. Post hoc tests did not reveal any difference in mean acceptance rates ON versus OFF for self or other separately (*p*s > 0.05). However, qualitative examination suggests the effect was driven by increased willingness to accept work offers for other when ON medication versus OFF, particularly when the effort required was greatest (Fig. 2*B*) and the amount of reward was higher (Fig. 2*C*), with no overall change in the self-condition (Fig. 2*A*). Thus, dopaminergic medication boosted the willingness to decide to work in the prosocial condition relative to the self-condition.

While previous studies have shown the effects of dopamine state on reward and effort sensitivity in PD (Chong et al., 2015; Manohar et al., 2015; Le Heron et al., 2018), there was no evidence that sensitivity to reward or effort changed significantly ON versus OFF medication (Table 2). In line with previous studies using this task in healthy populations (Lockwood et al., 2017), PD patients accepted more work offers when they required lower effort [OR = 0.27, SE = 0.06, CI: (0.18, 0.40), *z* = −6.25, *p *< 0.001] or larger reward [OR = 5.63, SE = 1.53, CI: (3.31, 9.58), *z* = 6.37, *p *< 0.001]. That is, patients were still showing a self-bias in motivation, being more willing to exert effort for themselves than others, and this was the case both ON and OFF medication (Fig. 3).

**Table 2.** Statistical results from a GLMM on choices to work versus rest, comparing patients ON and OFF dopaminergic medication

Parameter	Coefficient	SE	CI low	CI high	*z*	*p*
Intercept	99.64	53.42	34.84	284.96	8.58	<0.001
Drug	1.36	0.66	0.52	3.51	0.63	0.53
Recipient	2.45	0.59	1.53	3.91	3.75	<0.001
Reward	5.63	1.53	3.31	9.58	6.37	<0.001
Effort	0.27	0.06	0.18	0.40	−6.25	<0.001
Drug:recipient	0.74	0.09	0.59	0.93	−2.59	0.010
Drug:reward	1.24	0.35	0.71	2.14	0.76	0.45
Recipient:reward	1.14	0.16	0.87	1.49	0.96	0.33
Drug:effort	0.96	0.21	0.62	1.48	−0.20	0.84
Recipient:effort	1.03	0.07	0.90	1.17	0.37	0.71
Drug:recipient:reward	0.90	0.09	0.74	1.09	−1.09	0.27
Drug:recipient:effort	1.06	0.10	0.89	1.26	0.63	0.53

SE, standard error; CI, confidence interval.

Next, we ran several control analyses to establish the robustness of the finding that levodopa increases motivation to exert effort for another person, compared with for oneself. Although ON versus OFF drug was manipulated within-subject and session order was counterbalanced, it is possible that order effects could impact choice behavior. Incorporating session in the GLMM reported above did provide some evidence that session order modulated choices—a significant session:drug:recipient interaction [OR = 0.61, SE = 0.07, CI: (0.49, 0.77), *z* = −4.25, *p *< 0.001], with patients tested OFF medication first showing greater boosting of choices to work for others by medication, while participants tested ON medication first displayed less prominent changes in prosocial motivation. However, importantly, the drug:recipient interaction outlined above was still significant [OR = 0.74, SE = 0.09, CI: (0.59, 0.92), *z* = −2.64, *p *= 0.008] indicating that even though session order may impact the effect of medication on prosocial motivation, there is still an overall effect of dopamine state on prosocial versus self-choices.

Another possibility is that the interaction between medication state and recipient is driven by differences in successful executions of the chosen levels of force. However, overall success at the effortful exertions by PD patients was very high (OFF medication: 95.1%, ON: 94.5%, HC: 97.2%), with no significant difference in success comparing patients ON versus OFF levodopa (LMM of success *p *= 0.19), and success did not predict choice behavior in either drug state [OFF: *b* = 0.14 (−0.63, 0.91), *t* = 0.37, *p *= 0.71; ON: *b* = −0.17 (−0.83, 0.49), *t* = −0.53, *p *= 0.60]. Thus, success was very high, and failures at exerting a chosen effort did not predict choices behavior in PD patients. This suggests that decisions in all conditions were driven by an aversion to effort, rather than to risk or reward probability (Birnbaum, 2008; Contreras-Huerta et al., 2020).

### PD patients and healthy controls show a reduced willingness to exert effort for other versus self

Next, we tested for differences in choice behavior between healthy controls and patients OFF medication or healthy controls and patients ON medication. Across both contrasts, there were no significant differences between patients and controls in overall willingness to work or how recipient, effort, or reward affected choices (all *p*s > 0.05; see Tables 3 and 4 for full results). Moreover, there were no significant differences in credits obtained between patients and controls either ON or OFF medication (PDon vs PDoff, *W* = 345, *p *= 0.86; HC vs PDoff, *W* = 842, *p *= 0.52; HC vs PDon, *W* = 838, *p *= 0.56).

**Table 3.** Statistical results from a GLMM on choices to work versus rest, comparing patients OFF dopaminergic medication against healthy controls

Parameter	Coefficient	SE	CI low	CI upper	*z*	*p*
Intercept	81.67	37.40	33.28	200.37	9.61	<0.001
GroupPD off	1.44	0.95	0.40	5.25	0.55	0.58
Recipient	2.12	0.48	1.36	3.30	3.33	<0.001
Reward	7.82	2.15	4.57	13.40	7.49	<0.001
Effort	0.28	0.06	0.18	0.43	−5.85	<0.001
GroupPD off:recipient	1.37	0.43	0.74	2.53	1.00	0.32
GroupPD off:reward	0.82	0.32	0.38	1.77	−0.50	0.62
Recipient:reward	1.46	0.19	1.13	1.89	2.87	0.004
GroupPD off:rffort	0.97	0.31	0.52	1.81	−0.09	0.93
GroupPD off:recipient:reward	1.01	0.17	0.73	1.42	0.08	0.94
GroupPD off:recipient:effort	0.97	0.09	0.82	1.16	−0.29	0.77

SE, standard error; CI, confidence interval.

**Table 4.** Statistical results from a GLMM on choices to work versus rest, comparing patients ON dopaminergic medication against healthy controls

Parameter	Coefficient	SE	CI low	CI upper	*z*	*p*
Intercept	81.23	39.22	31.53	209.26	9.11	<0.001
GroupPD on	1.49	1.03	0.39	5.77	0.58	0.56
Recipient	1.88	0.41	1.23	2.87	2.90	0.004
Reward	7.30	2.16	4.08	13.05	6.70	<0.001
Effort	0.27	0.06	0.17	0.42	−5.71	<0.001
GroupPD on:recipient	0.96	0.27	0.55	1.68	−0.14	0.89
GroupPD on:reward	0.92	0.39	0.40	2.09	−0.20	0.84
Recipient:reward	1.32	0.17	1.02	1.70	2.11	0.034
GroupPD on:effort	0.94	0.32	0.49	1.82	−0.18	0.86
Recipient:effort	1.09	0.07	0.96	1.23	1.33	0.18
GroupPD on:recipient:reward	0.82	0.13	0.60	1.12	−1.25	0.21
GroupPD on:recipient:effort	1.00	0.09	0.83	1.20	0.01	0.99

SE, standard error; CI, confidence interval.

Like the patients, healthy controls were less willing to work when it benefitted another person [OR = 2.12, SE = 0.48, CI: (1.36, 3.30), *z* = 3.33, *p *< 0.001; Fig. 3*A*] or required more effort [OR = 0.28, SE = 0.06, CI: (0.18, 0.43), *z* = −5.85, *p *< 0.001; Fig. 3*C*] but more motivated to exert effort for higher rewards [OR = 7.82, SE = 2.15, CI: (4.57, 13.40), *z* = 7.49, *p *< 0.001; Fig. 3*D*]. Thus, the effect of dopaminergic medication in PD patients seemed to influence the difference in choices between self or other, but overall behavior was similar to controls.

### Patients incentivization by reward changes over trials and is impacted by medication

Previous research has shown that people's willingness to exert effort for reward declines over time during a task, which may be linked to fatigue and dopaminergic function (Iodice et al., 2017; Müller et al., 2021). We observed that incorporating trial number into the hypothesis-driven GLMMs better accounted for the observed data, resulting in superior model fit. In GLMMs comparing patients (both ON and OFF medication) to controls, a significant three-way interaction between trial number, reward, and group was observed [PDoff vs HC: OR = 0.54 (0.30, 0.97), SE = 0.16, *z* = −2.06, *p *= 0.039; PDon vs HC: OR = 0.57 (0.37, 0.89), SE = 0.13, *z* = −2.50, *p *= 0.012]. These interactions point toward differences in the ways rewards were viewed over the course of the experiment, with healthy participants demonstrating a relative reduction in the acceptance of low reward offers over time, while for patients this change over time was less pronounced.

In all three groups (PDoff, PDon, HC), significant interactions between effort and trial number showed participants chose fewer of the highest effort options over the course of the experiment [PDoff GLMM interaction: OR = 0.79 (0.69, 0.90), SE = 0.05, *z* = −3.56, *p *< 0.001; HC GLMM interaction: OR = 0.61 (0.47, 0.80), SE = 0.08, *z* = −3.54, *p *< 0.001; PDon post hoc interaction: *F* ratio = 36.90, *p *< 0.001]. Reward by trial number interactions also showed that unmedicated patients and healthy controls chose fewer of the lowest reward offers toward the end of the task [PDoff: OR = 1.23 (1.07, 1.41), SE = 0.09, *z* = 2.86, *p *= 0.004; HC: OR = 3.12 (1.97, 4.93), SE = 0.73, *z* = 4.87, *p *< 0.001; PDon post hoc interaction: *F* ratio = 3.33, *p *= 0.068]. In summary, both healthy controls and PD patients adapted their behavior over the task to prioritize options that required less effort or obtained larger rewards, with healthy controls prioritizing based on rewards to a greater extent than patients. Importantly, we did not find evidence of any three-way interactions between group/dopamine state, recipient, and trial number. Thus, there was no evidence of either accumulated effort or reward causing differences in inequity aversion-driven choices in our participant groups.

### Levodopa invigorates motor responses

**Figure 4.**
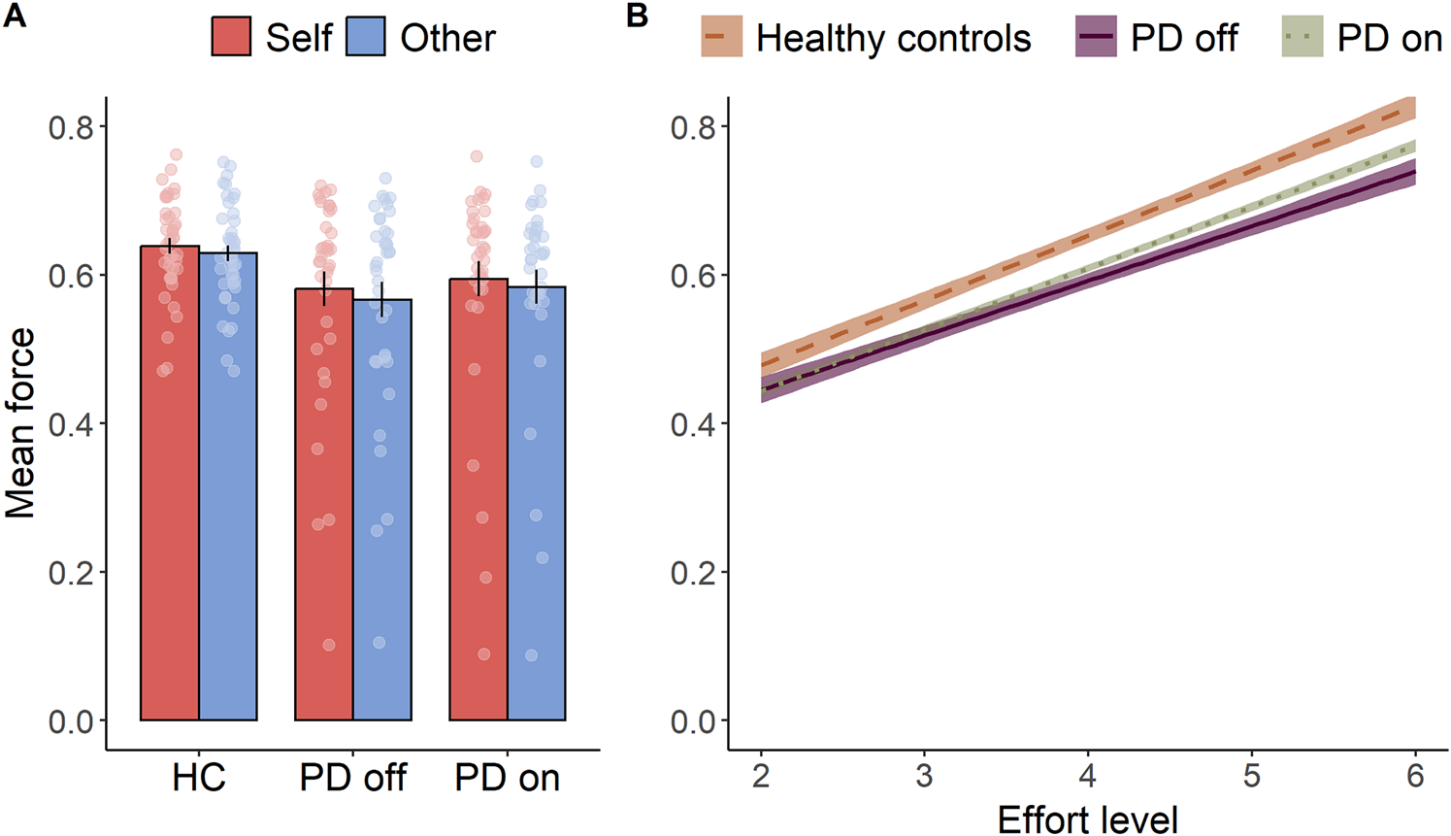
***A***, Mean force—area under the curve during the force period—exerted on trials where participants chose to work, collapsed across self, and other. ***B***, Force LM-estimated conditional means ± 95% confidence intervals across effort levels in the three groups (HC, PD ON, PD OFF).

After participants made a choice, they were then required to exert the force that they chose. Previous work suggests a degree to which people's choices don't align with their exertion of force. Specifically, even when people choose to help others, they exert less force into prosocial acts compared with self-benefitting ones (Lockwood et al., 2017). Moreover, dopaminergic medication is known to increase movement vigor (Beierholm et al., 2013; Le Bouc et al., 2016; Zénon et al., 2016). Thus, to examine whether dopamine administration influenced motor vigor in work trials, we performed LMMs on the force (defined as the area under the curve on each, with force scaled to a participant’s maximum area under the curve in each session of the experiment, controlling for overall differences in maximum force output), to examine effects of disease or medication state on the exertion of effort. When comparing patients ON versus OFF medication, no main effect of drug was observed [OR = 1.08, SE = 0.08, CI: (0.92, 1.26), *t* = 0.94, *p *= 0.35; Fig. 4*A*; Table 5].

**Table 5.** Statistical results from an LMM on force exerted (area under the curve of the force period) on trials where work was chosen, comparing patients ON versus OFF dopaminergic medication

Parameter	Coefficient	SE	CI low	CI upper	*t*	*p*
Intercept	0.98	0.12	0.77	1.25	−0.15	0.88
Drug	1.08	0.08	0.92	1.26	0.94	0.35
Recipient	1.02	0.01	1.00	1.04	2.58	0.010
Reward	1.04	0.01	1.02	1.06	3.59	<0.001
Effort	1.69	0.05	1.60	1.78	19.69	<0.001
Drug:recipient	0.99	0.01	0.97	1.00	−1.77	0.077
Drug:reward	1.00	0.01	0.97	1.03	0.07	0.94
Recipient:reward	1.00	0.01	0.99	1.01	−0.47	0.64
Drug:effort	1.05	0.02	1.01	1.10	2.55	0.011
Recipient:effort	1.00	0.01	0.99	1.02	0.86	0.39
Drug:recipient:reward	1.00	0.01	0.99	1.02	0.11	0.91
Drug:recipient:effort	0.99	0.01	0.98	1.01	−0.65	0.52

SE, standard error; CI, confidence interval.

There was a significant drug:effort interaction [OR = 1.05, SE = 0.02, CI: (1.01, 1.10), *t* = 2.55, *p *= 0.011; Fig. 4*B*], corresponding to greater force at higher-effort levels when ON medication, a finding replicated in similar studies in which dopamine has shown to invigorate motor responses (Zénon et al., 2016; Le Heron et al., 2018; Michely et al., 2020). There was also a significant main effect of reward [OR = 1.04, SE = 0.01, CI: (1.02, 1.06), *t* = 3.59, *p *< 0.001], with higher rewards leading to greater amounts of force produced, an effect observed in previous similar studies (Lockwood et al., 2017). Moreover, the LMM also demonstrated a significant effect of recipient [OR = 1.02, SE = 0.01, CI: (1.00, 1.04), *t* = 2.58, *p *= 0.010], indicating that patients exhibited superficial prosociality, investing less force in choices that resulted in benefit for another person compared with themselves. There was a trend for this to depend on medication state, but the drug:recipient interaction did not reach significance [OR = 0.99, SE = 0.01, CI: (0.97, 1.00), *t* = −1.77, *p *= 0.077].

Comparing healthy controls and patients OFF medication, a significant main effect of group was observed [OR = 0.71. SE = 0.10, CI: (0.53, 0.94), *t* = −2.40, *p *= 0.017; see Table 6 for full results], consistent with patients OFF medication producing significantly less force than controls. There was again a group:effort interaction [OR = 0.89, SE = 0.04, CI: (0.82, 0.96), *t* = −3.05, *p *= 0.002], with patients not exerting the same levels of relative force as healthy controls in tasks demanding greater expenditure of effort. Comparing healthy controls and patients ON medication, there was a trend toward less force in patients [OR = 0.77, SE = 0.11, CI: (0.58, 1.01), *t* = −1.88, *p *= 0.061; see Table 7 for full results], but otherwise the model indicated no significant differences between controls and patients. Participants’ maximum voluntary contraction, calibrated at the beginning of the experiment, importantly showed no overall differences between patients and controls (HC vs PDoff; *t* = 0.015, *p *= 0.99; HC vs PDon; *t* = −0.44, *p *= 0.66) or between patients ON and OFF medication (PDon vs PDoff; *t* = −1.23, *p *= 0.23). Overall, these results show that PD patients were less able to produce higher levels of force, even when this is adapted to an idiosyncratic MVC, when OFF medication compared with ON, with some evidence of a difference between patients and controls.

**Table 6.** Statistical results from an LMM on force exerted (area under the curve of the force period) on trials where work was chosen, comparing patients OFF versus healthy controls

Parameter	Coefficient	SE	CI low	CI upper	*t*	*p*
Intercept	1.21	0.12	1.00	1.47	1.97	0.049
GroupPD off	0.71	0.10	0.53	0.94	−2.40	0.017
Recipient	1.00	0.01	0.99	1.02	0.62	0.54
Reward	1.04	0.01	1.02	1.06	4.27	<0.001
Effort	2.01	0.06	1.91	2.12	25.51	<0.001
GroupPD off:recipient	1.02	0.01	1.00	1.03	1.83	0.067
GroupPD off:reward	1.00	0.01	0.98	1.03	0.26	0.79
Recipient:reward	0.99	0.01	0.98	1.01	−0.96	0.34
GroupPD off:effort	0.89	0.04	0.82	0.96	−3.05	0.002
Recipient:effort	1.00	0.01	0.99	1.01	−0.56	0.58
GroupPD off:recipient:reward	1.00	0.01	0.99	1.02	0.44	0.66
GroupPD off:recipient:effort	1.01	0.01	0.99	1.03	0.98	0.33

SE, standard error; CI, confidence interval.

**Table 7.** Statistical results from an LMM on force exerted (area under the curve of the force period) on trials where work was chosen, comparing patients ON versus healthy controls

Parameter	Coefficient	SE	CI low	CI upper	*t*	*p*
Intercept	1.16	0.11	0.96	1.40	1.56	0.12
GroupPD on	0.77	0.11	0.58	1.01	−1.88	0.061
Recipient	1.01	0.01	0.99	1.02	0.68	0.49
Reward	1.04	0.01	1.02	1.06	3.66	<0.001
Effort	1.99	0.06	1.88	2.11	23.13	<0.001
GroupPD on:recipient	1.00	0.01	0.98	1.02	0.18	0.86
GroupPD on:reward	1.00	0.02	0.98	1.03	0.32	0.75
Recipient:reward	0.99	0.01	0.98	1.00	−1.14	0.25
GroupPD on:effort	0.94	0.04	0.86	1.02	−1.48	0.14
Recipient:effort	1.00	0.01	0.99	1.01	−0.31	0.76
GroupPD on:recipient:reward	1.00	0.01	0.99	1.02	0.41	0.68
GroupPD on:recipient:effort	1.00	0.01	0.98	1.02	0.20	0.84

SE, standard error; CI, confidence Interval.

## Discussion

Every day we make decisions about whether to exert effort into prosocial acts that benefit others. However, whether dopamine is associated with such prosocial decisions is unknown. We tested the willingness to “work” and exert effort for self and for an anonymous other in PD patients tested ON and OFF their dopaminergic medication. We found an interaction between medication state and recipient, such that patients were more willing to choose to “work” for other compared with self, ON versus OFF dopamine. This effect could not be explained by the ability to exert the required force or order of medication effects.

Previous studies have painted a mixed picture on the effect of dopamine on prosocial behaviors. Boosting dopamine can increase inequity aversion (Sáez et al., 2015; Artigas et al., 2019), a seemingly prosocial behavior, and has been linked to increased generosity (Amstutz et al., 2021). However, other studies suggested that dopamine blockage can make people more prosocial (Soutschek et al., 2017) and dopamine-boosting medication can lead to increased selfishness (Pedroni et al., 2014; Crockett et al., 2015), with people more willing to profit from other's harm.

Our results provide evidence in support of the former set of studies, with increased levels of prosocial choices when dopamine is boosted. However, our results go beyond existing evidence by demonstrating an effect of dopamine when participants are neither trading off their own immediate rewards against someone else's, nor are they making this decision when the cost is financial as in many existing studies (Lockwood et al., 2020). Rather we show that dopamine boosts the willingness to exert effort for prosocial acts, which has not previously been demonstrated in PD patients or in healthy people.

In previous research, Parkinson's disease has consistently shown to be associated with lower willingness to invest physical effort for rewards, which is remediated by dopaminergic medication (Chong et al., 2015; Le Bouc et al., 2016; Le Heron et al., 2018). Similar effects have been observed in healthy participants undergoing pharmacological manipulation of dopamine (Wardle et al., 2011; Michely et al., 2020). It is therefore somewhat surprising that we did not find any difference in willingness to exert effort for self ON versus OFF medication in patients. In addition, it is highly unlikely that dopamine is a prosocially specific neuromodulator, given the extensive body of research examining dopamine's effects in nonsocial settings, so why might we have found an effect putatively modulating prosocial but not self-benefitting behaviors?

There are several possibilities: One is that boosting dopamine increases motivation to reach more abstract goals over more extended periods of time, rather than simply reducing the cost of effort or increasing the incentivization by rewards. Thus, if patients wish to be very prosocial, and that is their more abstract goal while performing this task, boosting dopamine may have boosted the motivation for this goal, translating into more choices to work in the prosocial condition. Such an interpretation is consistent with recent evidence showing that (1) dopamine neurons can change their firing in a context-dependent manner when the value of a higher-order goal is changed (Batten et al., 2024) and (2) can increase their firing when the value of reward is reduced when an animal is sated and there is no longer a goal to seek more of that reward (Papageorgiou et al., 2016; Han et al., 2021; Grove et al., 2022) and (3) that neural systems can flip how they value actions depending on one's goal (Frömer et al., 2019). All of this points to the possibility that dopamine may have a role in motivating behaviors that serve higher-order goals (in this case, working for others as well as oneself), rather than specific effects on the sensitivity to reward or effort.

Another possibility is that patients indeed found the costs of the effort higher OFF dopamine medication compared with ON and thus used “other” trials as a rest when OFF medication. Previous studies have consistently shown that dopamine depletion reduces incentivization by reward and heightens effort's costs (Chong et al., 2015; Manohar et al., 2015; Zénon et al., 2016; Le Heron et al., 2018; Pessiglione et al., 2018; Michely et al., 2020; Westbrook et al., 2020). A separate line of research suggests that motivation can be partially restored in effortful tasks very similar to this, albeit without a social condition, by choosing to rest (Müller et al., 2021; Matthews et al., 2023). Thus, it is plausible that patients were more selfish when OFF dopaminergic medication as they used the “other” trials as an opportunity to rest and ensure they still maximized reward for themselves. In that sense, they may appear more prosocial ON medication as they feel less need to rest and thus can work harder for others.

It is important to note that regardless of which interpretation is more accurate, the findings here point to dopamine depletion and medication having important impacts on prosocial behavior in everyday life in PD. Self-benefitting and prosocial choices are inherently intermixed in the real world (Contreras-Huerta et al., 2020), with ongoing behaviors often interrupted by opportunities to act prosocially or not (Gabay and Apps, 2021). As such, within the context of both explanations above, changes to dopamine levels will very likely have an impact on decisions to exert prosocial effort. Future research could use variations of the current design to directly compare these different explanations.

Notably, we found choices to “work” over the course of the experiment changed over trials between patients and controls. It is plausible that this is because of fatigue. It is well known that PD patients can suffer from higher levels of fatigue than healthy people (Havlikova et al., 2008; Sáez-Francàs et al., 2014), and although this may manifest as overall reductions in motivation, it might also be that this only develops dynamically during effortful tasks. Indeed it has recently been shown that the willingness to exert effort can change considerably over time during extended tasks as well as sensations of fatigue and that this may depend on dopamine levels (Iodice et al., 2017; Müller et al., 2021; Matthews et al., 2023). As such, patients may show different changes to their motivation ON or OFF medication over time, and compared with controls, due to a dynamic interplay between dopamine, sensations of fatigue, and the impact on motivation. While we designed our task to minimize the influence of fatigue by making sure participants did not encounter multiple high-effort options in a row, including breaks, and ensuring participants were never requested to exert force beyond 70% of their maximum, future studies could measure the influence of fatigue parametrically on self and prosocial motivation by using trial-by-trial subjective fatigue ratings or testing multiple clinical groups where fatigue is also common, such as in people with multiple sclerosis.

While this study provides important insights into the role of dopamine in the willingness to exert effort into prosocial acts, it is important to note some limitations to our conclusions. Firstly, we did not find any interaction effects between medication state, recipient, and either reward or effort level as has been found previously (Chong et al., 2015; Le Heron et al., 2018; McGuigan et al., 2019; Westbrook et al., 2020). This may have been because the extra conditions in the design (i.e., recipient of the reward) left us underpowered compared with previous studies, or it could be that dopamine simply increased a bias in the willingness to work and did not specifically change sensitivity to reward or effort (or exerted effects within the interaction of effort and reward, which the study was underpowered to test and therefore this interaction term wasn't included in mixed linear models). In addition, it is well known that neuromodulators are impacted in PD beyond dopamine (Maillet et al., 2016; Nobis et al., 2023), but in this study, we restricted our modulation to dopaminergic augmentation with medication that is a frontline treatment in PD. However, there is also evidence that both serotonin and noradrenaline are also depleted in PD. Both have been associated with reduced motivation or apathy in PD (Maillet et al., 2016; Hezemans et al., 2022) with effort-based decisions and the exertion of physical force (Varazzani et al., 2015; Husain and Roiser, 2018). In parallel, serotonin has also been implicated in social decision-making (Wood et al., 2006; Crockett et al., 2010, 2015; Bengart et al., 2021). Thus, future work should examine how other neurotransmitters influence prosocial motivation, including in PD. Lastly, it is important to note that we did not find any differences in controls in how willing patients were to put in effort for self or other, either ON or OFF dopamine, which could have related to similar (low) levels of apathy reported in both groups. While this means we cannot draw any strong links to differences in social behavior comparing PD to controls in existing research, it shows the effects we observed were specifically due to the dopaminergic manipulation in the patients. This highlights the importance of our within-subject repeated session design.

Here, we examined people's willingness to exert effort for reward when either oneself or another person can benefit. We show that PD patients ON dopamine medication are more willing to exert effort for others relative to self, compared with when they are OFF medication. This supports the idea that dopamine may play a role in motivating people to be more prosocial when trying to obtain rewards for self and other.

## Data Availability

Data will be made available at the time of publication.

## References

[B1] Aarsland D, Påhlhagen S, Ballard CG, Ehrt U, Svenningsson P (2011) Depression in Parkinson disease–epidemiology, mechanisms and management. Nat Rev Neurol 8:35–47. 10.1038/nrneurol.2011.189 22198405

[B2] Amstutz D, Michelis JP, Debove I, Maradan-Gachet ME, Lachenmayer ML, Muellner J, Schwegler K, Krack P (2021) Reckless generosity, Parkinson’s disease and dopamine: a case series and literature review. Mov Disord Clin Pract 8:469–473. 10.1002/mdc3.13156 33816681 PMC8015883

[B3] Ang Y-S, Lockwood P, Apps MAJ, Muhammed K, Husain M (2017) Distinct subtypes of apathy revealed by the apathy motivation index. PLoS One 12:e0169938. 10.1371/journal.pone.0169938 28076387 PMC5226790

[B4] Artigas SO, Liu L, Strang S, Burrasch C, Hermsteiner A, Münte TF, Park SQ (2019) Enhancement in dopamine reduces generous behaviour in women. PLoS One 14:e0226893. 10.1371/journal.pone.0226893 31891605 PMC6938376

[B5] Barr DJ, Levy R, Scheepers C, Tily HJ (2013) Random effects structure for confirmatory hypothesis testing: keep it maximal. J Mem Lang 68:255–278. 10.1016/j.jml.2012.11.001 PMC3881361 24403724

[B6] Batten SR, et al. (2024) Dopamine and serotonin in human substantia nigra track social context and value signals during economic exchange. Nat Hum Behav 8:718–728. 10.1038/s41562-024-01831-w 38409356 PMC11045309

[B7] Beierholm U, Guitart-Masip M, Economides M, Chowdhury R, Düzel E, Dolan R, Dayan P (2013) Dopamine modulates reward-related vigor. Neuropsychopharmacology 38:1495–1503. 10.1038/npp.2013.48 23419875 PMC3682144

[B8] Bengart P, Gruendler T, Vogt B (2021) Acute tryptophan depletion in healthy subjects increases preferences for negative reciprocity. PLoS One 16:e0249339. 10.1371/journal.pone.0249339 33784350 PMC8009398

[B9] Birnbaum MH (2008) New paradoxes of risky decision making. Psychol Rev 115:463–501. 10.1037/0033-295X.115.2.463 18426300

[B10] Chong TT-J, Bonnelle V, Manohar S, Veromann K-R, Muhammed K, Tofaris GK, Hu M, Husain M (2015) Dopamine enhances willingness to exert effort for reward in Parkinson’s disease. Cortex 69:40–46. 10.1016/j.cortex.2015.04.003 25967086 PMC4533227

[B11] Contreras-Huerta LS, Lockwood PL, Bird G, Apps MAJ, Crockett MJ (2022) Prosocial behavior is associated with transdiagnostic markers of affective sensitivity in multiple domains. Emotion 22:820–835. 10.1037/emo0000813 32718171 PMC9301775

[B12] Contreras-Huerta LS, Pisauro MA, Apps MAJ (2020) Effort shapes social cognition and behaviour: a neuro-cognitive framework. Neurosci Biobehav Rev 118:426–439. 10.1016/j.neubiorev.2020.08.003 32818580

[B13] Crockett MJ, Clark L, Lieberman MD, Tabibnia G, Robbins TW (2010) Impulsive choice and altruistic punishment are correlated and increase in tandem with serotonin depletion. Emotion 10:855–862. 10.1037/a0019861 21171757 PMC3009596

[B14] Crockett MJ, Siegel JZ, Kurth-Nelson Z, Ousdal OT, Story G, Frieband C, Grosse-Rueskamp JM, Dayan P, Dolan RJ (2015) Dissociable effects of serotonin and dopamine on the valuation of harm in moral decision making. Curr Biol 25:1852–1859. 10.1016/j.cub.2015.05.021 26144968 PMC4518463

[B15] Forbes PA, Aydogan G, Braunstein J, Todorova B, Wagner IC, Lockwood PL, Apps MA, Ruff CC, Lamm C (2023) Acute stress reduces effortful prosocial behaviour. Elife 12:RP87271. 10.7554/eLife.87271 PMC10942768 38180785

[B16] Forno LS (1996) Neuropathology of Parkinson’s disease. J Neuropathol Exp Neurol 55:259–272. 10.1097/00005072-199603000-00001 8786384

[B17] Frömer R, Dean Wolf CK, Shenhav A (2019) Goal congruency dominates reward value in accounting for behavioral and neural correlates of value-based decision-making. Nat Commun 10:4926. 10.1038/s41467-019-12931-x 31664035 PMC6820735

[B18] Gabay AS, Apps MA (2021) Foraging optimally in social neuroscience: computations and methodological considerations. Soc Cogn Affect Neurosci 16:782–794. 10.1093/scan/nsaa037 32232360 PMC8343566

[B19] Grove JC, Gray LA, Medina LS, La Santa Medina N, Sivakumar N, Ahn JS, Corpuz TV, Berke JD, Kreitzer AC, Knight ZA (2022) Dopamine subsystems that track internal states. Nature 608:374–380. 10.1038/s41586-022-04954-0 35831501 PMC9365689

[B20] Han Y, Xia G, He Y, He Y, Farias M, Xu Y, Wu Q (2021) A hindbrain dopaminergic neural circuit prevents weight gain by reinforcing food satiation. Sci Adv 7:eabf8719. 10.1126/sciadv.abf8719 34039606 PMC10964976

[B21] Hauser RA (2009) Levodopa: past, present, and future. Eur Neurol 62:1–8. 10.1159/000215875 19407449

[B22] Havlikova E, Rosenberger J, Nagyova I, Middel B, Dubayova T, Gdovinova Z, Groothoff JW, van Dijk JP (2008) Clinical and psychosocial factors associated with fatigue in patients with Parkinson’s disease. Parkinsonism Relat Disord 14:187–192. 10.1016/j.parkreldis.2007.07.017 17890136

[B23] Hezemans FH, et al. (2022) Noradrenergic deficits contribute to apathy in Parkinson’s disease through the precision of expected outcomes. PLoS Comput Biol 18:e1010079. 10.1371/journal.pcbi.1010079 35533200 PMC9119485

[B24] Husain M, Roiser JP (2018) Neuroscience of apathy and anhedonia: a transdiagnostic approach. Nat Rev Neurosci 19:470–484. 10.1038/s41583-018-0029-9 29946157

[B25] Iodice P, Ferrante C, Brunetti L, Cabib S, Protasi F, Walton ME, Pezzulo G (2017) Fatigue modulates dopamine availability and promotes flexible choice reversals during decision making. Sci Rep 7:535. 10.1038/s41598-017-00561-6 28373651 PMC5428685

[B26] Kawamura M, Koyama S (2007) Social cognitive impairment in Parkinson’s disease. J Neurol 254:IV49–IV53. 10.1007/s00415-007-4008-8

[B27] Le Bouc R, Rigoux L, Schmidt L, Degos B, Welter M-L, Vidailhet M, Daunizeau J, Pessiglione M (2016) Computational dissection of dopamine motor and motivational functions in humans. J Neurosci 36:6623–6633. 10.1523/JNEUROSCI.3078-15.2016 27335396 PMC6601748

[B28] Le Heron C, Apps MAJ, Husain M (2017) The anatomy of apathy: a neurocognitive framework for amotivated behavior. Neuropsychologia 118:54–67. 10.1016/j.neuropsychologia.2017.07.003 28689673 PMC6200857

[B29] Le Heron C, Plant O, Manohar S, Ang Y-S, Jackson M, Lennox G, Hu MT, Husain M (2018) Distinct effects of apathy and dopamine on effort-based decision-making in Parkinson’s disease. Brain 141:1455–1469. 10.1093/brain/awy110 29672668 PMC5917786

[B30] Lockwood PL, Abdurahman A, Gabay AS, Drew D, Tamm M, Husain M, Apps MAJ (2021) Aging increases prosocial motivation for effort. Psychol Sci 32:668–681. 10.1177/0956797620975781 33860711 PMC7611497

[B31] Lockwood PL, Apps MAJ, Chang SWC (2020) Is there a ‘social’ brain? Implementations and algorithms. Trends Cogn Sci 24:802–813. 10.1016/j.tics.2020.06.011 32736965 PMC7501252

[B32] Lockwood PL, Cutler J, Drew D, Abdurahman A, Jeyaretna DS, Apps MA, Husain M, Manohar SG (2024) Human ventromedial prefrontal cortex is necessary for prosocial motivation. Nat Human Behav 8:1403–1416. 10.1038/s41562-024-01899-4 38802539 PMC11272586

[B33] Lockwood PL, Hamonet M, Zhang SH, Ratnavel A, Salmony FU, Husain M, Apps MAJ (2017) Prosocial apathy for helping others when effort is required. Nat Hum Behav 1:0131. 10.1038/s41562-017-0131 28819649 PMC5555390

[B34] Lockwood PL, Wittmann MK, Nili H, Matsumoto-Ryan M, Abdurahman A, Cutler J, Husain M, Apps MAJ (2022) Distinct neural representations for prosocial and self-benefiting effort. Curr Biol 32:4172–4185.e7. 10.1016/j.cub.2022.08.010 36029773 PMC9616728

[B35] Maillet A, et al. (2016) The prominent role of serotonergic degeneration in apathy, anxiety and depression in de novo Parkinson’s disease. Brain 139:2486–2502. 10.1093/brain/aww162 27538418

[B36] Manohar SG, Chong TT-J, Apps MAJ, Batla A, Stamelou M, Jarman PR, Bhatia KP, Husain M (2015) Reward pays the cost of noise reduction in motor and cognitive control. Curr Biol 25:1707–1716. 10.1016/j.cub.2015.05.038 26096975 PMC4557747

[B37] Matthews J, Pisauro MA, Jurgelis M, Mueller T, Vassena E, Chong TT-J, Apps MA (2023) Computational mechanisms underlying the dynamics of physical and cognitive fatigue. Cognition 240:105603. 10.1016/j.cognition.2023.105603 37647742

[B38] Matuschek H, Kliegl R, Vasishth S, Baayen H, Bates D (2017) Balancing type I error and power in linear mixed models. J Mem Lang 94:305–315. 10.1016/j.jml.2017.01.001

[B39] McGuigan S, Zhou S-H, Brosnan MB, Thyagarajan D, Bellgrove MA, Chong TT-J (2019) Dopamine restores cognitive motivation in Parkinson’s disease. Brain 142:719–732. 10.1093/brain/awy341 30689734

[B40] Michely J, Viswanathan S, Hauser TU, Delker L, Dolan RJ, Grefkes C (2020) The role of dopamine in dynamic effort-reward integration. Neuropsychopharmacology 45:1448–1453. 10.1038/s41386-020-0669-0 32268344 PMC7360543

[B41] Müller T, Klein-Flügge MC, Manohar SG, Husain M, Apps MAJ (2021) Neural and computational mechanisms of momentary fatigue and persistence in effort-based choice. Nat Commun 12:4593. 10.1038/s41467-021-24927-7 34321478 PMC8319292

[B42] Narme P, Mouras H, Roussel M, Duru C, Krystkowiak P, Godefroy O (2013) Emotional and cognitive social processes are impaired in Parkinson’s disease and are related to behavioral disorders. Neuropsychology 27:182–192. 10.1037/a0031522 23527646

[B43] Nobis L, Maio MR, Saleh Y, Manohar S, Kienast A, McGann E, Husain M (2023) Role of serotonin in modulation of decision-making in Parkinson’s disease. J Psychopharmacol 37:420–431. 10.1177/02698811221144636 36628992 PMC10101180

[B44] Papageorgiou GK, Baudonnat M, Cucca F, Walton ME (2016) Mesolimbic dopamine encodes prediction errors in a state-dependent manner. Cell Rep 15:221–228. 10.1016/j.celrep.2016.03.031 27050518 PMC4835543

[B45] Pedroni A, Eisenegger C, Hartmann MN, Fischbacher U, Knoch D (2014) Dopaminergic stimulation increases selfish behavior in the absence of punishment threat. Psychopharmacology 231:135–141. 10.1007/s00213-013-3210-x 23900641

[B46] Pell MD, Monetta L, Rothermich K, Kotz SA, Cheang HS, McDonald S (2014) Social perception in adults with Parkinson’s disease. Neuropsychology 28:905–916. 10.1037/neu0000090 24885448

[B47] Pessiglione M, Vinckier F, Bouret S, Daunizeau J, Le Bouc R (2018) Why not try harder? Computational approach to motivation deficits in neuro-psychiatric diseases. Brain 141:629–650. 10.1093/brain/awx278 29194534

[B48] Remy P, Doder M, Lees A, Turjanski N, Brooks D (2005) Depression in Parkinson’s disease: loss of dopamine and noradrenaline innervation in the limbic system. Brain 128:1314–1322. 10.1093/brain/awh445 15716302

[B49] Reniers RLEP, Corcoran R, Drake R, Shryane NM, Völlm BA (2011) The QCAE: a questionnaire of cognitive and affective empathy. J Pers Assess 93:84–95. 10.1080/00223891.2010.528484 21184334

[B50] Sáez-Francàs N, Hernández-Vara J, Corominas-Roso M, Alegre J, Jacas C, Casas M (2014) Relationship between poor decision-making process and fatigue perception in Parkinson’s disease patients. J Neurol Sci 337:167–172. 10.1016/j.jns.2013.12.003 24351900

[B51] Sáez I, Zhu L, Set E, Kayser A, Hsu M (2015) Dopamine modulates egalitarian behavior in humans. Curr Biol 25:912–919. 10.1016/j.cub.2015.01.071 25802148 PMC4627633

[B52] Salamone JD, Correa M, Farrar A, Mingote SM (2007) Effort-related functions of nucleus accumbens dopamine and associated forebrain circuits. Psychopharmacology 191:461–482. 10.1007/s00213-006-0668-9 17225164

[B53] Schapira AHV, Chaudhuri KR, Jenner P (2017) Non-motor features of Parkinson disease. Nat Rev Neurosci 18:435–450. 10.1038/nrn.2017.62 28592904

[B54] Sockeel P, Dujardin K, Devos D, Denève C, Destée A, Defebvre L (2006) The Lille apathy rating scale (LARS), a new instrument for detecting and quantifying apathy: validation in Parkinson’s disease. J Neurol Neurosurg Psychiatry 77:579–584. 10.1136/jnnp.2005.075929 16614016 PMC2117430

[B55] Soutschek A, et al. (2017) The dopaminergic reward system underpins gender differences in social preferences. Nat Hum Behav 1:819–827. 10.1038/s41562-017-0226-y 31024122

[B56] Sparrow EP, Leung R, Statucka M, Spaniol J, Cohn M (2021) Altruism in Parkinson’s disease. Neuropsychology 35:547–555. 10.1037/neu0000740 33939451

[B57] Varazzani C, San-Galli A, Gilardeau S, Bouret S (2015) Noradrenaline and dopamine neurons in the reward/effort trade-off: a direct electrophysiological comparison in behaving monkeys. J Neurosci 35:7866–7877. 10.1523/JNEUROSCI.0454-15.2015 25995472 PMC6795183

[B58] Wardle MC, Treadway MT, Mayo LM, Zald DH, de Wit H (2011) Amping up effort: effects of d-amphetamine on human effort-based decision-making. J Neurosci 31:16597–16602. 10.1523/JNEUROSCI.4387-11.2011 22090487 PMC3234999

[B59] Westbrook A, van den Bosch R, Määttä JI, Hofmans L, Papadopetraki D, Cools R, Frank MJ (2020) Dopamine promotes cognitive effort by biasing the benefits versus costs of cognitive work. Science 367:1362–1366. 10.1126/science.aaz5891 32193325 PMC7430502

[B60] Wood RM, Rilling JK, Sanfey AG, Bhagwagar Z, Rogers RD (2006) Effects of tryptophan depletion on the performance of an iterated prisoner’s dilemma game in healthy adults. Neuropsychopharmacology 31:1075–1084. 10.1038/sj.npp.1300932 16407905

[B61] Yesavage JA, Sheikh JI (1986) 9/Geriatric depression scale (GDS): recent evidence and development of a shorter version. Clin Gerontol 5:165–173. 10.1300/J018v05n01_09

[B62] Zénon A, Devesse S, Olivier E (2016) Dopamine manipulation affects response vigor independently of opportunity cost. J Neurosci 36:9516–9525. 10.1523/JNEUROSCI.4467-15.2016 27629704 PMC6601940

